# Di-2-pyridyl disulfide–succinic acid (1/1)

**DOI:** 10.1107/S1600536809016213

**Published:** 2009-05-07

**Authors:** Cheng Zhang, Ling Yuan, Ji-Yong Liu, Wei Xu

**Affiliations:** a State Key Laboratory Base of Novel Functional Materials and Preparation Science, Faculty of Materials Science and Chemical Engineering, Institute of Solid Materials Chemistry, Ningbo University, Ningbo, Zhejiang 315211, People’s Republic of China

## Abstract

In the title compound, C_10_H_8_N_2_S_2_·C_4_H_6_O_2_, both components of the cocrystal lie on crystallographic twofold rotation axes. In the di-2-pyridyl disulfide mol­ecule, the dihedral angle between the two pyridine rings is 66.6 (1)°. In the crystal structure, inter­molecular O—H⋯N and weak C—H⋯O hydrogen bonds link both types of mol­ecules into columns along the *c* axis.

## Related literature

For general background to the design of cocrystals, see: Desiraju (2003 ▶); Thalladi *et al.* (2007 ▶). For a related structure, see: Raghavan *et al.* (1977 ▶).
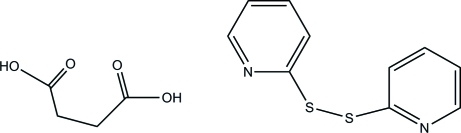


## Experimental

### 

#### Crystal data

C_10_H_8_N_2_S_2_·C_4_H_6_O_4_
*M* *_r_* = 338.39
Monoclinic, 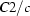

*a* = 8.4211 (17) Å
*b* = 13.347 (3) Å
*c* = 14.141 (3) Å
β = 98.43 (3)°
*V* = 1572.2 (6) Å^3^
*Z* = 4
Mo *K*α radiation
μ = 0.36 mm^−1^
*T* = 293 K
0.60 × 0.47 × 0.23 mm

#### Data collection

Rigaku R-AXIS RAPID diffractometer
Absorption correction: multi-scan (*ABSCOR*; Higashi, 1995 ▶) *T*_min_ = 0.822, *T*_max_ = 0.921
7089 measured reflections
1799 independent reflections
1490 reflections with *I* > 2σ(*I*)
*R*_int_ = 0.039

#### Refinement

*R*[*F* ^2^ > 2σ(*F* ^2^)] = 0.053
*wR*(*F* ^2^) = 0.139
*S* = 1.28
1799 reflections
128 parameters
H atoms treated by a mixture of independent and constrained refinement
Δρ_max_ = 0.40 e Å^−3^
Δρ_min_ = −0.30 e Å^−3^

### 

Data collection: *RAPID-AUTO* (Rigaku, 1998 ▶); cell refinement: *RAPID-AUTO*; data reduction: *CrystalStructure* (Rigaku/MSC, 2004 ▶); program(s) used to solve structure: *SHELXS97* (Sheldrick, 2008 ▶); program(s) used to refine structure: *SHELXL97* (Sheldrick, 2008 ▶); molecular graphics: *ORTEPII* (Johnson, 1976 ▶); software used to prepare material for publication: *SHELXL97*.

## Supplementary Material

Crystal structure: contains datablocks global, I. DOI: 10.1107/S1600536809016213/lh2792sup1.cif

Structure factors: contains datablocks I. DOI: 10.1107/S1600536809016213/lh2792Isup2.hkl

Additional supplementary materials:  crystallographic information; 3D view; checkCIF report

## Figures and Tables

**Table 1:** Hydrogen-bond geometry (Å, °)

*D*—H⋯*A*	*D*—H	H⋯*A*	*D*⋯*A*	*D*—H⋯*A*
O2—H2*H*⋯N1	0.83 (4)	1.94 (4)	2.759 (3)	173 (4)
C2—H2*A*⋯O1^i^	0.93	2.47	3.128 (4)	127

Symmetry code: (i) 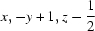.

**Fig. 1.**
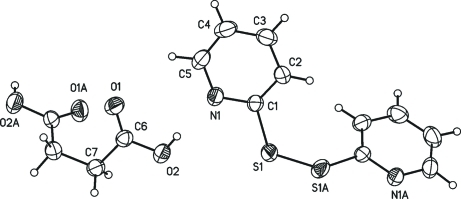
The molecular structure of the title compound with dispalcement ellipsoids drawn at the 45% probability level. The complete molecules of di-2-pyridyl disulfide and succinic acid are generated by the symmetry operators (-x, y, -z+1/2) and (-x, y, -z+3/2) respectively.

**Fig. 2.**
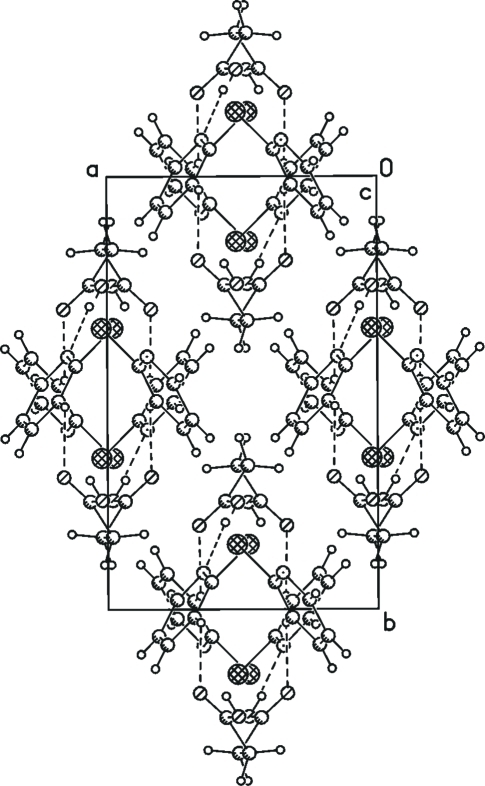
Part of the crystal structure of the title compound with hydrogen bonds shown as dashed lines.

## supplementary crystallographic information

### Comment

The design of cocrystals has been a field of intensive research in recent years. With reliable design strategies, cocrystals could offer a modular approach to developing materials with desirable properties. (Desiraju, 2003; Thalladi *et al.*, 2007). Weak noncovalent interactions such as hydrogen bonds are utilized to create cocrystals. Herein we report the structure of the title cocrystal.

The formula unit of the title compound (Fig. 1) contains one molecule of di-2-pyridyl disulfide (dpds) and one molecule succinic acid. The dihedral angle between the two pyridine rings of the dpds molecule is 66.6 (1)°, and the S—S bond length, 2.025 (2) Å, is not significantly different than that found in the structure of the free ligand, 2.016 (2) Å (Raghavan *et al.*, 1977). The torsion angle of the C6-C7-C7^ii^-C6^ii^ [symmetry code: (ii) -x, y, -z+3/2] backbone of succinic acid is 74.5 (3)°. The proton of the carboxylate O atom (O2) of the succinic acid molecule forms a strong hydrogen bond with atom N1 of the dpds molecule (see Table 1 for hydrogen bond geometry). In addition, in the crystal structure, weak intermolecular C-H···O hydrogen bonds supplement intermolecular N-H···O hydrogen bonds to form columns running parallel to the c-axis (Fig 2).

### Experimental

All chemicals were reagent grade quality obtained from commercial sources and without further purification. Dpds (0.2206 g, 1 mmol) and succinic acid (0.1181 g, 1 mmol) were dissolved in a H_2_O/EtOH solution (*v*/*v* = 2:1, 15 ml), which was stirred for 0.5 h and then filtrated, the filtrate was allowed to concentrate by slow evaporation to give colorless block crystals.

### Refinement

H atoms bonded to C atoms were palced in geometrically calculated positions (C-H = 0.93-0.97Å) and were refined in a riding-model approximation, with *U*_iso_(H) = 1.2 *U*_eq_(C). The H atom bonded to O2 atoms was located in a difference Fourier map and its position refined with *U*_iso_(H) = 1.5 *U*eq(O).

### Crystal data

**Table:**

C_10_H_8_N_2_S_2_·C_4_H_6_O_4_	*F*(000) = 704
*M**_r_* = 338.39	*D*_x_ = 1.430 Mg m^−^^3^
Monoclinic, *C*2/*c*	Mo *K*α radiation, λ = 0.71073 Å
Hall symbol: -C 2yc	Cell parameters from 4565 reflections
*a* = 8.4211 (17) Å	θ = 3.1–27.5°
*b* = 13.347 (3) Å	µ = 0.36 mm^−^^1^
*c* = 14.141 (3) Å	*T* = 293 K
β = 98.43 (3)°	Block, colorless
*V* = 1572.2 (6) Å^3^	0.60 × 0.47 × 0.23 mm
*Z* = 4	

### Data collection

**Table:**

Rigaku R-AXIS RAPID diffractometer	1799 independent reflections
Radiation source: fine-focus sealed tube	1490 reflections with *I* > 2σ(*I*)
graphite	*R*_int_ = 0.039
Detector resolution: 0 pixels mm^-1^	θ_max_ = 27.5°, θ_min_ = 3.1°
ω scans	*h* = −10→10
Absorption correction: multi-scan (*ABSCOR*; Higashi, 1995)	*k* = −17→17
*T*_min_ = 0.822, *T*_max_ = 0.921	*l* = −18→16
7089 measured reflections	

### Refinement

**Table:**

Refinement on *F*^2^	Primary atom site location: structure-invariant direct methods
Least-squares matrix: full	Secondary atom site location: difference Fourier map
*R*[*F*^2^ > 2σ(*F*^2^)] = 0.053	Hydrogen site location: inferred from neighbouring sites
*wR*(*F*^2^) = 0.139	H atoms treated by a mixture of independent and constrained refinement
*S* = 1.28	*w* = 1/[σ^2^(*F*_o_^2^) + (0.0282*P*)^2^ + 3.2667*P*] where *P* = (*F*_o_^2^ + 2*F*_c_^2^)/3
1799 reflections	(Δ/σ)_max_ < 0.001
128 parameters	Δρ_max_ = 0.40 e Å^−^^3^
0 restraints	Δρ_min_ = −0.30 e Å^−^^3^

### Special details

**Table:**

Geometry. All e.s.d.'s (except the e.s.d. in the dihedral angle between two l.s. planes) are estimated using the full covariance matrix. The cell e.s.d.'s are taken into account individually in the estimation of e.s.d.'s in distances, angles and torsion angles; correlations between e.s.d.'s in cell parameters are only used when they are defined by crystal symmetry. An approximate (isotropic) treatment of cell e.s.d.'s is used for estimating e.s.d.'s involving l.s. planes.
Refinement. Refinement of *F*^2^ against ALL reflections. The weighted *R*-factor *wR* and goodness of fit *S* are based on *F*^2^, conventional *R*-factors *R* are based on *F*, with *F* set to zero for negative *F*^2^. The threshold expression of *F*^2^ > σ(*F*^2^) is used only for calculating *R*-factors(gt) *etc*. and is not relevant to the choice of reflections for refinement. *R*-factors based on *F*^2^ are statistically about twice as large as those based on *F*, and *R*- factors based on ALL data will be even larger.

### Fractional atomic coordinates and isotropic or equivalent isotropic displacement parameters (Å^2^)

**Table:**

	*x*	*y*	*z*	*U*_iso_*/*U*_eq_	
N1	−0.1474 (3)	0.58448 (17)	0.44834 (16)	0.0396 (6)	
C1	−0.1175 (3)	0.56306 (19)	0.36047 (19)	0.0349 (6)	
C2	−0.1815 (4)	0.4813 (2)	0.3079 (2)	0.0436 (7)	
H2A	−0.1580	0.4692	0.2466	0.052*	
C3	−0.2816 (4)	0.4183 (2)	0.3497 (3)	0.0518 (8)	
H3A	−0.3273	0.3627	0.3165	0.062*	
C4	−0.3132 (4)	0.4382 (2)	0.4408 (3)	0.0540 (8)	
H4A	−0.3792	0.3961	0.4702	0.065*	
C5	−0.2449 (4)	0.5218 (2)	0.4873 (2)	0.0474 (7)	
H5A	−0.2673	0.5356	0.5484	0.057*	
S1	0.02142 (10)	0.65005 (6)	0.32235 (6)	0.0473 (3)	
C6	−0.0647 (3)	0.7501 (2)	0.63801 (19)	0.0362 (6)	
C7	0.0187 (4)	0.8324 (2)	0.69918 (19)	0.0395 (6)	
H7A	−0.0128	0.8965	0.6699	0.047*	
H7B	0.1337	0.8254	0.7008	0.047*	
O1	−0.1622 (3)	0.69379 (16)	0.66296 (15)	0.0513 (6)	
O2	−0.0193 (3)	0.74777 (17)	0.55229 (15)	0.0523 (6)	
H2H	−0.065 (5)	0.701 (3)	0.521 (3)	0.079*	

### Atomic displacement parameters (Å^2^)

**Table:**

	*U*^11^	*U*^22^	*U*^33^	*U*^12^	*U*^13^	*U*^23^
N1	0.0424 (14)	0.0434 (13)	0.0345 (12)	0.0021 (11)	0.0106 (10)	0.0002 (9)
C1	0.0338 (15)	0.0335 (13)	0.0394 (14)	0.0023 (11)	0.0122 (11)	0.0003 (10)
C2	0.0500 (19)	0.0368 (15)	0.0474 (17)	−0.0022 (13)	0.0184 (13)	−0.0075 (12)
C3	0.055 (2)	0.0339 (15)	0.069 (2)	−0.0067 (14)	0.0180 (16)	−0.0032 (14)
C4	0.053 (2)	0.0447 (17)	0.069 (2)	−0.0020 (15)	0.0237 (16)	0.0166 (15)
C5	0.0502 (19)	0.0556 (18)	0.0399 (16)	0.0046 (15)	0.0177 (13)	0.0109 (13)
S1	0.0518 (5)	0.0440 (4)	0.0515 (5)	−0.0135 (4)	0.0255 (4)	−0.0117 (3)
C6	0.0394 (16)	0.0363 (14)	0.0330 (13)	0.0031 (12)	0.0057 (11)	0.0053 (10)
C7	0.0424 (16)	0.0387 (14)	0.0372 (15)	−0.0043 (12)	0.0054 (12)	0.0025 (11)
O1	0.0617 (15)	0.0510 (13)	0.0432 (12)	−0.0169 (11)	0.0140 (10)	0.0003 (9)
O2	0.0669 (16)	0.0549 (14)	0.0390 (12)	−0.0156 (12)	0.0205 (10)	−0.0064 (9)

### Geometric parameters (Å, °)

**Table:**

N1—C1	1.334 (3)	C5—H5A	0.9300
N1—C5	1.345 (4)	S1—S1^i^	2.0251 (17)
C1—C2	1.385 (4)	C6—O1	1.204 (3)
C1—S1	1.787 (3)	C6—O2	1.324 (3)
C2—C3	1.384 (4)	C6—C7	1.507 (4)
C2—H2A	0.9300	C7—C7^ii^	1.516 (5)
C3—C4	1.380 (5)	C7—H7A	0.9700
C3—H3A	0.9300	C7—H7B	0.9700
C4—C5	1.378 (5)	O2—H2H	0.83 (4)
C4—H4A	0.9300		
			
C1—N1—C5	117.2 (3)	N1—C5—H5A	118.5
N1—C1—C2	123.9 (3)	C4—C5—H5A	118.5
N1—C1—S1	111.3 (2)	C1—S1—S1^i^	106.10 (10)
C2—C1—S1	124.8 (2)	O1—C6—O2	123.7 (3)
C3—C2—C1	117.6 (3)	O1—C6—C7	124.6 (3)
C3—C2—H2A	121.2	O2—C6—C7	111.7 (2)
C1—C2—H2A	121.2	C6—C7—C7^ii^	113.6 (2)
C4—C3—C2	119.7 (3)	C6—C7—H7A	108.8
C4—C3—H3A	120.2	C7^ii^—C7—H7A	108.8
C2—C3—H3A	120.2	C6—C7—H7B	108.8
C5—C4—C3	118.5 (3)	C7^ii^—C7—H7B	108.8
C5—C4—H4A	120.7	H7A—C7—H7B	107.7
C3—C4—H4A	120.7	C6—O2—H2H	109 (3)
N1—C5—C4	123.1 (3)		

Symmetry codes: (i) −*x*, *y*, −*z*+1/2; (ii) −*x*, *y*, −*z*+3/2.

### Hydrogen-bond geometry (Å, °)

**Table:**

*D*—H···*A*	*D*—H	H···*A*	*D*···*A*	*D*—H···*A*
O2—H2H···N1	0.83 (4)	1.94 (4)	2.759 (3)	173 (4)
C2—H2A···O1^iii^	0.93	2.47	3.128 (4)	127

Symmetry codes:  (iii) *x*, −*y*+1, *z*−1/2.
